# A GPBAR1 (TGR5) Small Molecule Agonist Shows Specific Inhibitory Effects on Myeloid Cell Activation *In Vitro* and Reduces Experimental Autoimmune Encephalitis (EAE) *In Vivo*

**DOI:** 10.1371/journal.pone.0100883

**Published:** 2014-06-26

**Authors:** Nuruddeen D. Lewis, Lori A. Patnaude, Josephine Pelletier, Donald J. Souza, Susan M. Lukas, F. James King, Jonathan D. Hill, Dimitria E. Stefanopoulos, Kelli Ryan, Sudha Desai, Donna Skow, Stefan G. Kauschke, Andre Broermann, Daniel Kuzmich, Christian Harcken, Eugene R. Hickey, Louise K. Modis

**Affiliations:** 1 Departments of Immunology and Inflammation, Boehringer Ingelheim Pharmaceuticals Inc., Ridgebury, Connecticut, United States of America; 2 Research Networking, Boehringer Ingelheim Pharmaceuticals Inc., Ridgebury, Connecticut, United States of America; 3 CardioMetabolic Diseases Research, Boehringer Ingelheim Pharmaceuticals Inc., Biberach, Germany; 4 Medicinal Chemistry, Boehringer Ingelheim Pharmaceuticals Inc., Ridgebury, Connecticut, United States of America; Friedrich-Alexander University Erlangen, Germany

## Abstract

GPBAR1 is a G protein-coupled receptor that is activated by certain bile acids and plays an important role in the regulation of bile acid synthesis, lipid metabolism, and energy homeostasis. Recent evidence suggests that GPBAR1 may also have important effects in reducing the inflammatory response through its expression on monocytes and macrophages. To further understand the role of GPBAR1 in inflammation, we generated a novel, selective, proprietary GPBAR1 agonist and tested its effectiveness at reducing monocyte and macrophage activation in vitro and in vivo. We have used this agonist, together with previously described agonists to study agonism of GPBAR1, and shown that they can all induce cAMP and reduce TLR activation-induced cytokine production in human monocytes and monocyte-derived macrophages in vitro. Additionally, through the usage of RNA sequencing (RNA-Seq), we identified a select set of genes that are regulated by GPBAR1 agonism during LPS activation. To further define the in vivo role of GPBAR1 in inflammation, we assessed GPBAR1 expression and found high levels on circulating mouse monocytes. Agonism of GPBAR1 reduced LPS-induced cytokine production in mouse monocytes ex vivo and serum cytokine levels in vivo. Agonism of GPBAR1 also had profound effects in the experimental autoimmune encephalomyelitis (EAE) mouse model of multiple sclerosis, where monocytes play an important role. Mice treated with the GPBAR1 agonist exhibited a significant reduction in the EAE clinical score which correlated with reduced monocyte and microglial activation and reduced trafficking of monocytes and T cells into the CNS. These data confirm the importance of GPBAR1 in controlling monocyte and macrophage activation in vivo and support the rationale for selective agonists of GPBAR1 in the treatment of inflammatory diseases.

## Introduction

GPBAR1 (TGR5) is a G-protein coupled receptor that is activated by bile acids. GPBAR1 and other bile acid receptors contribute to bile acid homeostasis, which is critical for absorption of dietary lipids and fat-soluble vitamins, and the elimination of excess cholesterol. GPBAR1 is expressed in a variety of tissues including the spleen and brown adipose tissue as well as in monocytes, and in enteroendocrine cells in the intestine where its activation improves glucose homeostasis, insulin sensitivity, and increases energy expenditure [1]. Consequently, development of specific GPBAR1 agonists for the treatment of diabetes and obesity has been an area of very active research [2], [3].

Recent work in vitro using natural bile acids (e.g. oleanolic acid, an agonist for both GPBAR1 and FXR receptors [4]) suggests an additional role for these receptors in blunting macrophage responses to TLR activation, resulting in reduced proinflammatory cytokine production and phagocytosis. This effect has been demonstrated in vitro using human cells and has also been observed in other species [5]–[9] and it is consistent with GPBAR1 expression in human immune cells (particularly on CD14^+^ monocytes) [10]. GPBAR1 activation with bile acids in myeloid cells mediates cAMP induction, activation of PKA, and phosphorylation of CREB [10], [11] leading to reduced NFκB signaling [12].

Studies with mice null for the GPBAR1 receptor suggest that it has a role in macrophage activation in vivo; GPBAR1^−/−^ mice treated with LPS had abundant macrophage infiltration into the liver and enhanced liver pathology compared to wild-type controls [12] and GPBAR1^−/−^ mice also had more severe injury-induced colitis than wild-type controls [13]. Consistent with these observations, treatment of wild-type mice with oleanolic acid reduced disease severity and monocyte infiltration into the colon [13] and reduced disease severity in a model of EAE [14]. Additionally, GPBAR1^−/−^ LDLR^−/−^ mice have increased macrophage activation and lesion formation when on a high fat diet in a model of atherosclerosis [15]. As above, bile acids could protect against this macrophage activation in the wild-type GPBAR1 background. While these studies are suggestive of GPBAR1 agonism having the potential to be a pharmacological intervention point for the treatment of inflammatory disorders, it remains to be shown that a *selective* GPBAR1 agonist is sufficient to mediate anti-inflammatory effects, both in vitro and in vivo, in pharmacological models of inflammation and that the observed pharmacological effects to date with non-selective bile acids at high concentrations are not driven through alternative bile acid receptors such as FXR.

We therefore generated a selective, potent GPBAR1 agonist and evaluated its potential to reduce macrophage activation in vitro and in vivo with a view to developing it as a novel therapeutic agent for the treatment of inflammatory disorders. We confirmed published reports of GPBAR1 induction of cAMP and its blockade of LPS-mediated cytokine production in primary human monocytes and macrophages. We extended the current knowledge around GPBAR1 signaling in human myeloid cells by evaluating additional proinflammatory stimuli and by performing RNA-Seq on monocytes in vitro, where we observed a selective impact of GPBAR1 agonism on a subset of genes that were changed by LPS stimulation. Importantly, we demonstrated that the selective GPBAR1 agonist reduced disease severity in EAE, the mouse model of multiple sclerosis where monocyte activity is critical. The effect on disease correlated with decreased activation of both monocytes and microglia and reduced numbers of monocytes and T cells in the CNS. These data confirm the ability of GPBAR1 agonist to control monocyte activation in vivo and support the rationale for exploring the role of agonism of GPBAR1 in inflammatory diseases.

## Methods

All experiments using human blood samples and animals were performed under protocols approved by the Boehringer-Ingelheim Institutional Animal Care and Use Committee and according to the United States Animal Welfare Act.

### Human monocyte and macrophage assays

Human peripheral blood mononuclear cells isolated with leukapheresis were purchased from AllCells (Alameda, CA), frozen in 80% FBS, 10% RPMI and 10% DMSO and stored in liquid nitrogen until used. Primary human CD14^+^/CD16^+^ monocytes were isolated by negative selection (StemCell Technologies kit 19058, CD14^+^ without CD16^+^ depletion) and used directly or differentiated to macrophages in the presence of human M-CSF (R&D Systems, 10 ng/ml) for 7 days on collagen IV-coated microtiter plates (BD Biosciences). Five independent donors were used for the experiments described in this manuscript, and several of these donors were assayed in duplicate, triplicate or quadruplicate for each experiment as indicated in the figure legends. Monocytes were used at a density of 25,000 cells per well in a 96-well plate in RPMI, 10% FBS, penicillin/streptomycin (monocytes) and 10 ng/ml M-CSF (macrophages). They were either used directly or differentiated for 7 days, prior to priming with 100 ng/ml of IFNγ followed by stimulation with one of the proinflammatory stimuli for 24 hours: 100 pg/ml LPS, (TLR4 agonist, *Escherichia coli* 0111:B4, Sigma), 1000 ng/ml PAM3CSK4 (TLR1/2), 10^8^ cells/ml HKLM (TLR2), 1000 ng/ml Poly I:C (TLR3), 100 ng/ml Flagellin (TLR5), 10 ng/ml FSL-1 (TLR6/2), 100 ng/ml Imiquimod (TLR7), 1000 ng/ml ssRNA (TLR8), 500 nM ODN2006 (TLR9) (all from Invivogen), 250 ng/ml TNFα (R&D Systems), 250 ng/ml Mega CD40L (Enzo), 40 ng/ml IL-1β (R&D Systems). Compounds were dissolved to 10 mM in DMSO, tested at 0.1% DMSO, and were pre-incubated with cells for 30 minutes prior to the addition of any stimuli. Levels of cytokine in the supernatants were determined by sandwich immunoassay (TNFα and IL-12p40, MesoScale Discovery, as individual analytes or in a multiplex between IL-12p40, IL-6, IL-1β and TNFα proinflammatory 39-plex, MILLIPLEX MAP Human Cytokine/Chemokine Magnetic Bead Panel, HCYTOMAG-60K proinflammatory, Millipore for BioRad FlexMap 3D. Levels of TNFα or IL-12p40 (in pg/ml) were determined based on the standard curve provided in the kits. Stimuli alone in the presence of DMSO without compound were used as positive controls and no stimuli as negative controls. Negative controls were performed in every experiment where the cells were incubated with vehicle (DMSO at the same concentration as used in the presence of compounds/treatment). IC_50_ values were calculated using XLfit (IDBS) with a 4-parameter fit.

### cAMP assays

CHO cells expressing human GPBAR1 were generated by transfecting T-Rex CHO cells, which stably express the Tet repressor by lipofection with constructs based on pT-Rex-DEST30 with the coding sequence for human GPBAR1 and selection with Geneticin (400 µg/ml) to identify stable cell clones. Using the reference compound EX00000246, compound C [16], expression and activation of the GPBAR1 receptor could be demonstrated in a tetracycline- and GPBAR1 agonist-dependent manner. The performance of the GPBAR1-expressing T-Rex CHO cell line was stable for at least 40 cell passages. The parental, non-transfected T-Rex CHO cell line showed no response to GPBAR1 agonists (data not shown). CHO cells expressing human GPBAR1 were plated at a density of 5,000 cells per well in DMEM:F12 mixed in a 1∶1 ratio, 15 mM HEPES, 2.5 mM Glutamax, and 10% FCS for 20 hours at 37°C. The media was removed prior to addition of agonists. Agonists (including either compounds or natural ligands such as Lithocholic acid (Sigma)) were diluted into KRBH stimulation buffer (130 mM NaCl, 3.6 mM KCl, 0.5 mM NaH_2_PO_4_, 0.5 mM MgSO_4_, 1.5 mM CaCl_2_, 10 mM HEPES, 5 mM NaHCO_3_, 10 mM NaCl, 0.5 mM IBMX, 0.1% BSA) and added to the wells. The cells were incubated at 23°C (room temperature, to facilitate automation and place handling) for 45 minutes. The assay was stopped with the addition of cAMP detection reagents (AlphaScreen, PerkinELmer) and was read on a PE Envision (AlphaScreen mode). 1000 nM of EX00000246 was used as a positive control and wells containing no agonist where the cells were incubated with vehicle (DMSO at the same concentration as used in the presence of compounds/treatment) were used as negative controls and were performed in every experiment. All compounds tested were subjected to a 10 point dose response curve in 1% DMSO. cAMP assays were performed in duplicate on 2 separate occasions. EC_50_ values were calculated using XLfit (IDBS) with a 4-parameter fit.

### Off-target selectivity profile of GPBAR1 agonists

The off-target profiles of BIX02694 and EX00000246 were assessed in a selectivity screen at a standard concentration of 10 µM and tested in duplicate (Eurofins Panlabs, Taipei, Taiwan). The methods specific to each assay performed can be found at www.eurofinspanlabs.com/Panlabs using the assay number listed in parentheses after each assay: Adenosine A1 (200510), Adenosine A2A (200610), Adrenergic α1A (203100), Adrenergic α1B (203200), Adrenergic β1 (204010), Adrenergic β2 (204110), Calcium Channel L-Type, Dihydropyridine site (214600), Cannabinoid CB1 (217030), Dopamine D1 (219500), Dopamine D2S (219700), GABAA, Flunitrazepam, central (226600), GABAA, Muscimol, Central (226500), Glutamate, NMDA, Phencyclidine (233000), Histamine H1 (239610), Imidazoline I2, Central (241000), Muscarinic M2 (252710), Muscarinic M3 (252810), Nicotinic Acetylcholine (258590), Nicotinic Acetylcholine α1, Bungarotoxin (258700), Norepinephrine Transporter (NET; 204410), Opiate μ (260410), Phorbol Ester (264500), Potassium Channel, KATP (265600), Potassium Channel, hERG (265900), Prostanoid EP4 (268420), Rolipram (270000), Serotonin 5-HT2B (271700), Sigma σ1 (278110), Sodium Channel, Site 2 (279510), Androgen (testosterone) AR (285010), Estrogen ERα (226010), Estrogen ERβ (226050), Progesterone (268000), Retinoid X Receptor RXRα (327147), Thyroid Hormone (285900), Vitamin D3 (288010), Farnesoid X Receptor (311600), Liver X Receptor alpha (331810), Liver X Receptor beta (331820), Peroxisome Proliferator Activated Receptor alpha (338200), Peroxisome Proliferator Activated Receptor γ,(338250), and Retinoic Acid Receptor α (338600).

### mRNA expression analysis

Taqman PCR was performed on mouse whole blood and mouse leukocytes for both *Gpbar1* and 18s mRNA levels. Primer and probe sets were purchased from Life Technologies, probe IDs MM00558112_s1 and 4333760F, respectively. *Gpbar1* mRNA levels were also assessed on various immune cells (isolated by positive selection using EasySep kits from stem cell technologies for CD14+ monocytes N = 2 donors, CD3+T cells N = 6 donors, CD19+B cells N = 2 donors, NK cells N = 2 donors and isolated by negative selection, Neutrophils N = 3 donors) using GeneChip Human Genome U133 Plus 2.0 arrays as performed by Genelogic probe ID - 1552501_a_at. Probe intensities for 62 control probes (recommended by GeneExpress for the U133Plus2 chip were log transformed and subjected to quantile normalization and distribution scaling around the mean. Normalized values for Gpbar1 were then compared across the different cell types.

Human PBMCs isolated with leukapheresis were purchased from AllCells, frozen in 80% FBS, 10% RPMI, 10% DMSO and stored in liquid nitrogen until used. Primary human CD14^+^/CD16^+^ monocytes were isolated by negative selection (StemCell Technologies) and used directly. Cells were used at a density of 500,000 cells per well in RPMI, 10% FBS, penicillin/streptomycin. Compounds were dissolved to 10 mM in DMSO and tested at 500 nM in 0.1% DMSO. Compounds were pre-incubated with cells for 30 minutes prior to the addition of 100 ng/ml IFNγ and 100vpg/ml LPS (*Escherichia coli* 0111:B4, Sigma). At either 6 or 24 hours the cells were lysed directly in the well with RLT buffer (Qiagen) and RNA was isolated with the RNAeasy kit (Qiagen).

### RNA sequencing

The extracted RNA was put through 52vbp paired-end RNA-seq on the Illumina platform. Reads were aligned to the human genome (hg19) using STAR (version 2.3) [17]. Quantification of expression levels was performed with Cufflinks 2, while differential analysis between experimental conditions was performed using HTSeq and LIMMA [18]. Gene lists were selected from LIMMA results as overlapping sets of genes at each time point differentially regulated by stimulation (2 fold change, FDR-corrected p<0.05), and also regulated through treatment with EX00000246 (FDR p<0.05). These lists were further divided based on whether the influence of the compound was in the same or opposite direction as the response to the initial stimulus. The dataset can be found in the Gene Expression Omnibus: http://www.ncbi.nlm.nih.gov/geo/, accession numbers GSM1383738 through GSM1383777.

### Flow cytometry and intracellular cytokine staining on peripheral blood

Mouse blood was collected retroorbitally from female C57BL/6 mice for mRNA analysis, flow cytometry and ex vivo LPS stimulation. For mRNA analysis, blood was either lysed directly or after red blood cell lysis (Ammonium-Chloride-Potassium lysing buffer, Life Technologies) followed by RNA isolation using the RNeasy Mini kit (Qiagen) according to the manufacturer's instructions.

For ex vivo LPS stimulation, blood was plated at 50 µl per well. BIX02694 was diluted and added to the cells at the indicated concentrations. After 30 minutes, LPS was added at 100 ng/ml with brefeldin A (BioLegend). The total volume was 100 µl. The cells were incubated at 37°C for 5 hours, placed on ice and stained with antibodies against CD11b, Ly6G, CD115, and Ly6C for 20 minutes. Cells were then treated with 1X Lyse/Fix solution (eBiosciences) for 30 minutes followed by two washes, permeabilized with Wash/Perm solution (BD Biosciences) and stained with anti-TNFα antibody (BioLegend) for 20 minutes. Cells were washed twice and analyzed on a FACS Canto II.

### In vivo LPS challenge

To measure changes in LPS-induced serum cytokines in response to GPBAR1 agonists, female Balb/c mice weighing approximately 20 grams were purchased from Charles River Laboratories. Mice were orally dosed with vehicle (0.5% natrosol), Prednisolone (3 mg/kg), or BIX02694 one hour before being challenged with an intraperitoneal injection of 2 µg of LPS (E. coli 055:B5), n = 10 mice per group. Blood was collected retroorbitally at 1 hour (for TNFα measurement) and 4 hours (for IL-12p40 and CCL2 measurement) post-challenge and assessed for serum cytokines by sandwich immunoassay (MesoScale Discovery) or for monocyte production of TNFα by flow cytometry.

### EAE induction

EAE was induced as described [19]. Female C57BL/6 mice were subcutaneously injected with 100 µg of MOG35–55 (myelin oligodendrocyte glycoprotein; AnaSpec) in IFA (incomplete Freund's adjuvant; Difco) containing 250 µg of *Mycobacterium tuberculosis* (Difco). On the same day and again 48 hours later, mice received an intraperitoneal injection of 200 ng of pertussis toxin (List Biological Laboratories). Mice were dosed twice daily with BIX02694 or with vehicle starting the day before immunization until day 30. 100% disease incidence was observed in the vehicle control group compared to 70% incidence in the agonist treated animals (n = 10 per group). Mice were scored, blinded, as follows: 0, no symptoms; 1, limp tail; 2, limp tail and inability to right itself when placed on its back; 3, partial hind limb paralysis; 4, complete hind limb paralysis; 5, complete hind limb paralysis with forelimb involvement or moribund. Animals were monitored daily and given fluids (1 ml saline, subcutaneously) if signs of dehydration were observed and as a routine if they reached a score of 4. Animals were euthanized using isofluorane to induce anesthesia, followed by cervical dislocation and opening the diaphragm.

### Statistics

For the EAE clinical score data, area under the curve (AUC) was calculated using GraphPad Prism 5.04. The Mann-Whitney test was performed using the AUC data to determine significance. For other data, either an unpaired t-test was used when comparing two groups or a one-way ANOVA followed by the Newman-Keuls post test for comparison between multiple groups.

### Isolation and flow cytometry of CNS cells

Mice were placed under anesthesia and were perfused intracardially with PBS until the liver became blanched. The brain and spinal cord were homogenized in HBSS using a dounce homogenizer and cells were purified using a percoll gradient. Percoll PLUS (GE) (in DPBS) was added to the homogenate to a final concentration of 30% and overlayed onto a 70% percoll solution for centrifugation at 18°C for 20 minutes at 1500 rpm. The top layer of debris was removed and the cellular layer at the 30%/70% interphase was collected, washed and resuspended in cell staining buffer containing TruStain FcX (BioLegend) and incubated on ice for 15 minutes. Cells were then stained with antibodies for flow cytometry including Ly6C (HK1.4), CD115 (AFS98); CD86 (GL-1), I-A/I-E (M5/114.15.2), CD40 (3/23), CD69 (H1.2F3), CD80 (16-10A1), CD44 (IM7), CD62L (MEL-14). GPBAR1 protein expression was assessed on mouse blood cells by flow cytometry with an anti-GPBAR1 polyclonal antibody (clone: C-16; Santa Cruz Biotechnology).

## Results

**Figure 1:**
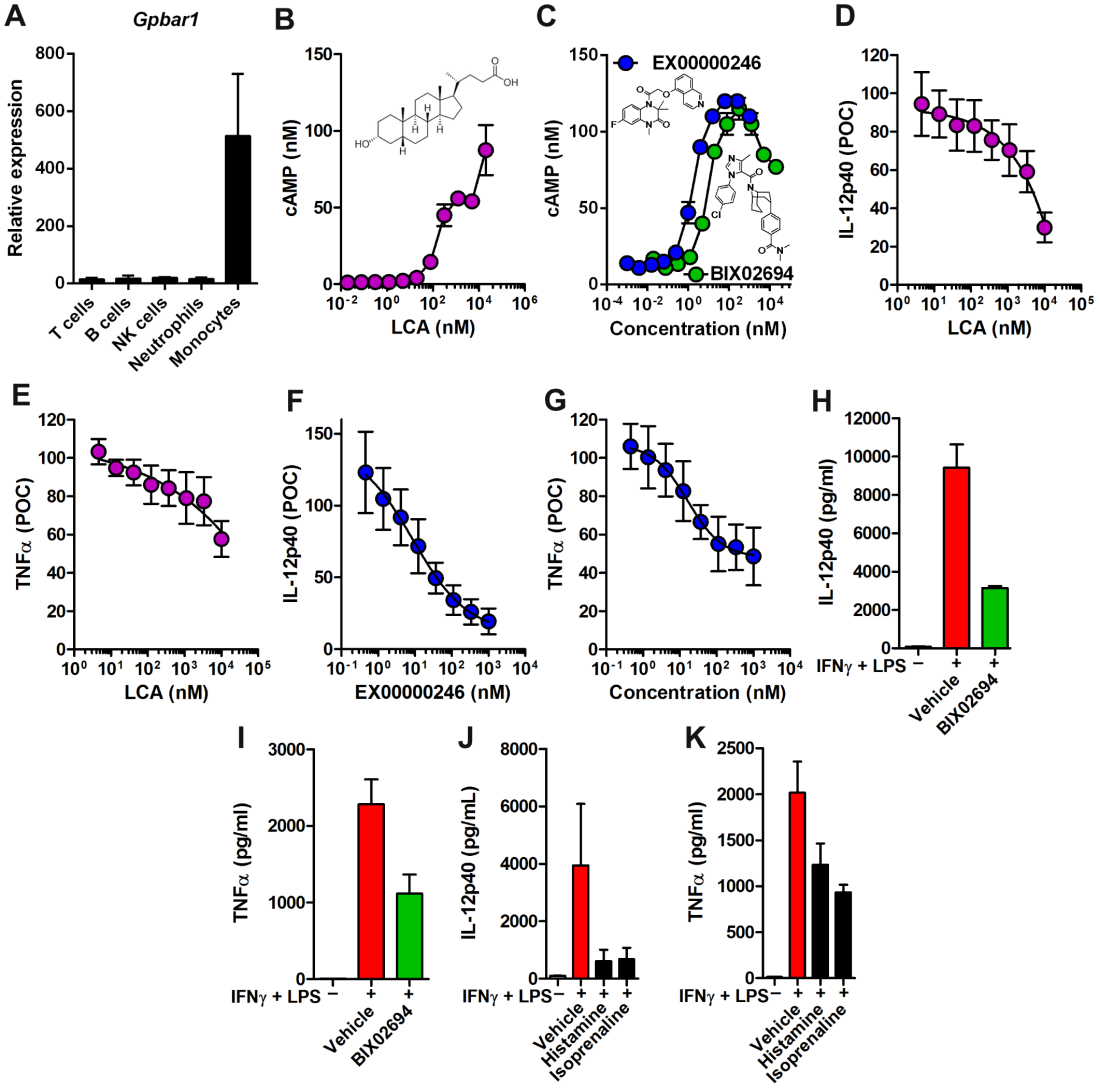
Agonism of GPBAR1 on human monocytes reduces proinflammatory cytokine production and enhances cAMP. (A) Immune cell populations (CD3^+^ T cells, CD19^+^ B cells, CD56^+^ NK cells, Neutrophils and CD14^+^ monocytes) were enriched from blood and *Gpbar1* mRNA levels were assessed by microarray using Affymetrix chip technology. Absolute expression is normalized to relative expression of 62 control probes across the entire chip. CHO cells expressing human GPBAR1 were generated and stimulated with GPBAR1 agonists Lithocholic acid (B) or EX00000246 and BIX02694 (C) and cAMP production was assessed. The chemical structures of the molecules are shown in each case. Human monocytes (4 donors, assayed in triplicate) were isolated by negative selection and pre-treated with increasing concentrations of Lithocholic acid (LCA) or EX00000246 or BIX02694 at 1 µM for 30 minutes before stimulation with IFNγ + LPS and data is presented as percent of control (POC) (D–G) or pg/ml (H, I). Controls with no ligand were all treated with 0.1% DMSO in media. Supernatants were collected after 24 hours and assessed for IL-12p40 (D, F, H) and TNFα (E, G, I) by a sandwich immunoassay. Production of IL-12p40 (J) and TNFα (K) by LPS-stimulated monocytes was assessed in the presence of histamine and isoprenaline at 1 µM. Protein levels of IL-12p40 and TNFα were assessed after 24 hours.

To understand the role of GPBAR1 in inflammation, we first confirmed that human monocytes express high levels of GPBAR1 as reported [10]. We assessed mRNA levels of *Gpbar1* by gene expression profiling using the Affymetrix platform in monocytes and compared it to other circulating immune cells. Monocytes expressed high levels of *Gpbar1* and we found that *Gpbar1* mRNA levels were low in T cells, B cells, NK cells, and neutrophils (Fig. 1A).

We next generated a CHO cell line expressing human GPBAR1 that shows increases in cAMP [10] as measured by an AlphaScreen cAMP assay kit, in response to a known GPBAR1 agonist, Lithocholic acid [10] (EC_50_ = 1 µM; Fig. 1B). We next identified multiple GPBAR1 agonists from our 890,000 compound library, such as BIX02694 (synthesized in-house) and other compounds (not shown), which induced dose-dependent increases in cAMP EC_50_ 9 nM (Fig. 1C) in a manner comparable to a reported GPBAR1 agonist EX00000246 (EC50 = 2 nM; Fig. 1C) [16]. These GPBAR1 agonists do not induce cAMP in the parental CHO cell line that does not express GPBAR1 (data not shown). We used these GPBAR1 agonists to confirm and extend the reported in vitro activities using human monocytes and macrophages.

We tested the ability of these GPBAR1 agonists, Lithocholic acid and EX00000246, to inhibit LPS-induced proinflammatory cytokine production in human monocytes and found dose-dependent reductions in IL-12p40 and TNFα in four donors (Fig. 1D–G). At a saturating concentration of 1 µM, IL-12p40 levels were reduced by >70% (inflection point  = 9 nM) and TNFα levels were reduced by approximately 50% (inflection point  = 16 nM). BIX02694 has limited solubility in the 0.1% DMSO vehicle tolerated in the monocyte and macrophage assays and therefore it is shown at a single high concentration (1 µM) in this assay format in Fig. 1H and 1I where it can reduce IL-12p40 production by approximately 60% and TNFα production by approximately 50%. These findings demonstrate that agonism of GPBAR1 reduces LPS-induced production of IL-12p40 and TNFα in human monocytes and confirm that the compounds used here have similar biological profiles to Lithocholic acid used here and bile acids used in previous studies [3]–[5]. BIX02694 and EX00000246 were tested against a panel of thirty-six receptors, ion channels, nuclear receptors, and other protein targets for off-target inhibition *via* radioligand binding. Additionally, twelve cellular assays were utilized to measure effects on nuclear hormone receptors. Neither compound elicited significant responses in any of the off-target assays (≥50% inhibition or stimulation at 10 µM, data not shown). Notably, the compounds tested had no significant displacement of radioligand binding at muscarinic M2 and M3 receptors, both known to interact with bile acids. To confirm that induction of cAMP can dampen proinflammatory responses, we tested if histamine and isoprenaline, two compounds that are also known to induce cAMP independently of GPBAR1, would have similar effects to a GPBAR1 agonist in decreasing proinflammatory cytokine production in monocytes [20], [21]. Both histamine and isoprenaline reduced the production of IL-12p40 and TNFα in LPS-stimulated monocytes (Fig. 1G and 1H), confirming previous reports that cAMP inducers can block proinflammatory cytokine production.

**Figure 2:**
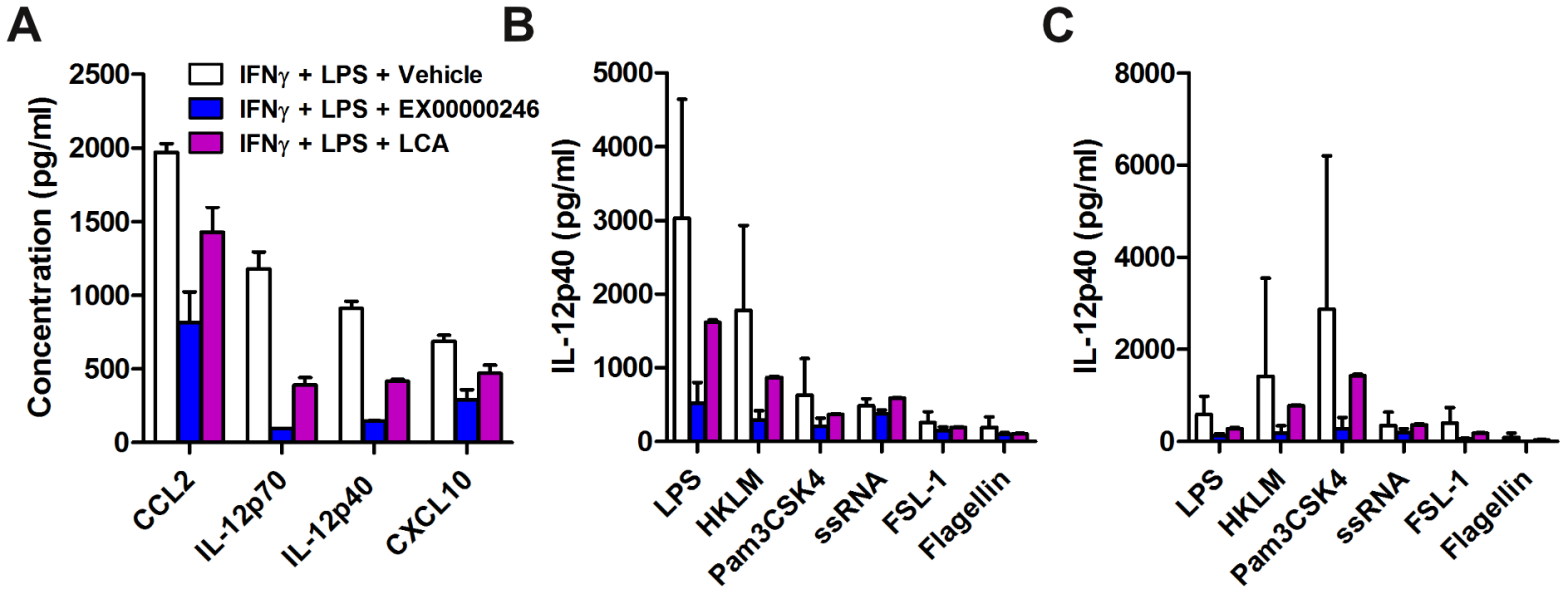
GPBAR1 agonism reduces cytokine production induced by various TLR ligands. (A) Production of cytokines at 24 hrs by LPS-stimulated monocytes (1 donor, quadruplicate) was assessed in the presence of EX00000246 (500 nM) or Lithocholic acid (10 µM) by BioRad FlexMap 3D technology using a multi-plex kit purchased from Millipore.Human monocytes (B) and monocyte-derived macrophages (C) (4 donors, assayed in triplicate) were stimulated with various TLR ligands in the presence of EX00000246 and production of IL-12p40 as measured by MSD was assessed at 24 hrs. Untreated negative controls reported cytokine levels at or below the detection limit and therefore are not plotted in these graphs.

In order to understand the extent of the impact of GPBAR1 agonism on blockade of LPS responsive proinflammatory cytokines, we assessed the levels of 39 cytokines and chemokines in the supernatants of LPS-stimulated human monocytes from a single donor in quadruplicate (Fig 2A) as a profiling experiment. We verified that the levels of IL-12p40 measured with this method was equivalent to the levels of IL-12p40 detected using the MSD methodology used throughout the rest of this work (data not shown). In addition to IL-12p40 shown in Figure 1, we found that GPBAR1 agonism (with Lithocholic acid at 10 µM and EX00000246 at 500 nM) also reduced CCL2, IL-12p70, and CXCL10 (Fig. 2A). Notably, GPBAR1 agonism did not limit LPS induced IL-1β and IL-6 production. This was confirmed using this donor and 3 additional donors (in triplicate) with a 4-plex MSD assay kit for IL-12p40, TNFα, IL-1β, and IL-6 (data not shown).

Next, we investigated whether reduced cytokine production with GPBAR1 agonism is restricted to TLR4 signaling (LPS stimulation). We stimulated human monocytes and macrophages (4 donors, in triplicate) with various TLR agonists and measured IL-12p40 in response to Lithocholic acid at 10 µM and EX00000246 at 500 nM (Fig 2B and 2C).The reduced level of inhibition by Lithocholic acid can be attributed to its lower potency compared to EX00000246. In addition to LPS, and similar to results shown by Haselow *et al* [5], we found that GPBAR1 agonism also restricted IL-12p40 production upon stimulation with heat-killed *Listeria monocytogenes*, a TLR2 agonist, and Pam3CSK4, a TLR1/2 agonist (Fig. 2B). We also tested monocyte-derived macrophages and found that although they reached high levels of activation from different TLR agonists, GPBAR1 agonism still reduced IL-12p40 levels (Fig. 2C). Although the induction of IL-12p40 is much lower in both monocytes and macrophages with FSL-1 (TLR6/2) and Flagellin (TLR5), GPBAR1 agonism still has an impact in reducing cytokine production. The exception is ssRNA (TLR8) where no reduction in cytokine induction is observed in either cell type. In all of these experiments, the cytokine production in the absence of stimulation was below the limit of detection and therefore not represented on the graphs. Taken together, these data demonstrate that GPBAR1 agonism induces intracellular cAMP and reduces the production of proinflammatory cytokines in human monocytes and macrophages and has a broad effect on dampening responses to different proinflammatory stimuli.

**Figure 3:**
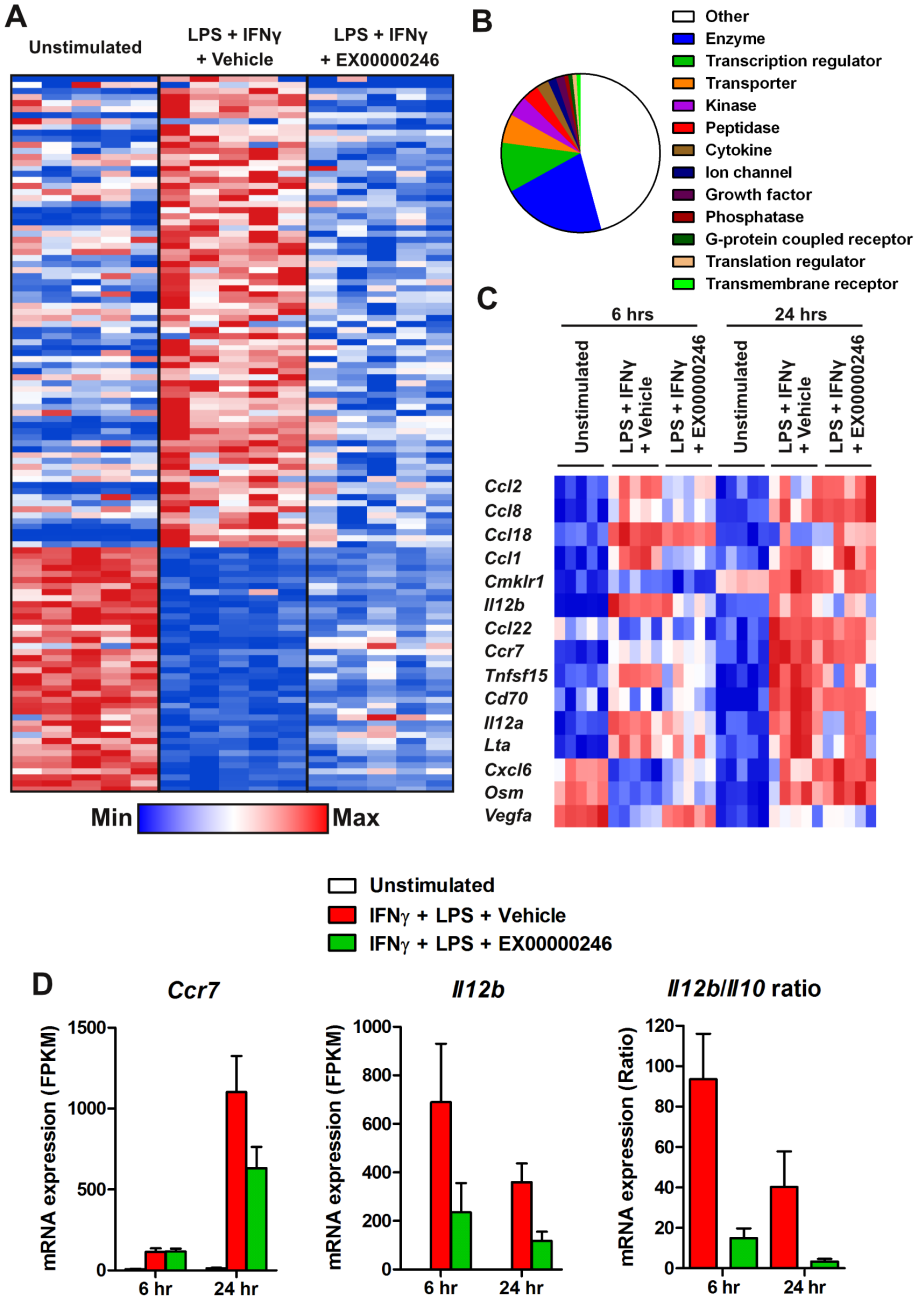
Agonism of GPBAR1 on human monocytes modulates a subset of LPS activated genes and blocks M1 differentiation. Monocytes from 5 donors were primed with IFNγ and stimulated with LPS ± agonist. RNA was isolated from these samples and subjected to RNA-Seq. (A) Heatmap of genes whose activation or repression by LPS at 6 hours was inhibited by the GPBAR1 agonist EX00000246. (B) Cellular function data from Ingenuity Systems was mapped onto the GPBAR1 regulated genes. (C) Heatmap of genes annotated as cytokine or chemokine whose activation or repression by LPS at 6 hours, 24 hours, or both times was inhibited by the GPBAR1 agonist EX00000246. (D) Individual plots of *Ccr7*, *Il12b* expression and ratio of *Il12/Il10* expression after stimulation with LPS treated with EX00000246.

To understand the impact of GPBAR1 agonism on monocytes beyond cytokine production, we analyzed gene expression changes by RNA-Seq after proinflammatory stimulation of peripheral monocytes from five independent donors using LPS with and without agonism of GPBAR1 for 6 hours and 24 hours. LPS stimulation led to modulation (both up and down, >2-fold change) of >1000 genes at 6 hours and 24 hours. A subset of these genes was modulated by GPBAR1 agonism (Fig. 3A). The genes whose responsiveness to LPS and IFNγ were modulated represent many cellular functions in addition to classic proinflammatory signals (Fig. 3B). Interestingly, a relatively small set of cytokines and chemokines were modulated by GPBAR1 inhibition at either 6 hours or 24 hours (Fig. 3C). Of these modulated cytokines, IL-12p40 is the only cytokine whose expression was blocked with inhibition at both timepoints. These results are consistent with our cytokine production results where we observed impacts on CCL2 and IL-12 (Fig. 2D). To understand the impact of GPBAR1 agonism on macrophage differentiation, we found that CCR7 and IL-12 upregulation is partially blocked and the IL-12:IL-10 ratio of production was decreased by treatment with an agonist (Fig. 3D), consistent with other reports [6].

**Figure 4:**
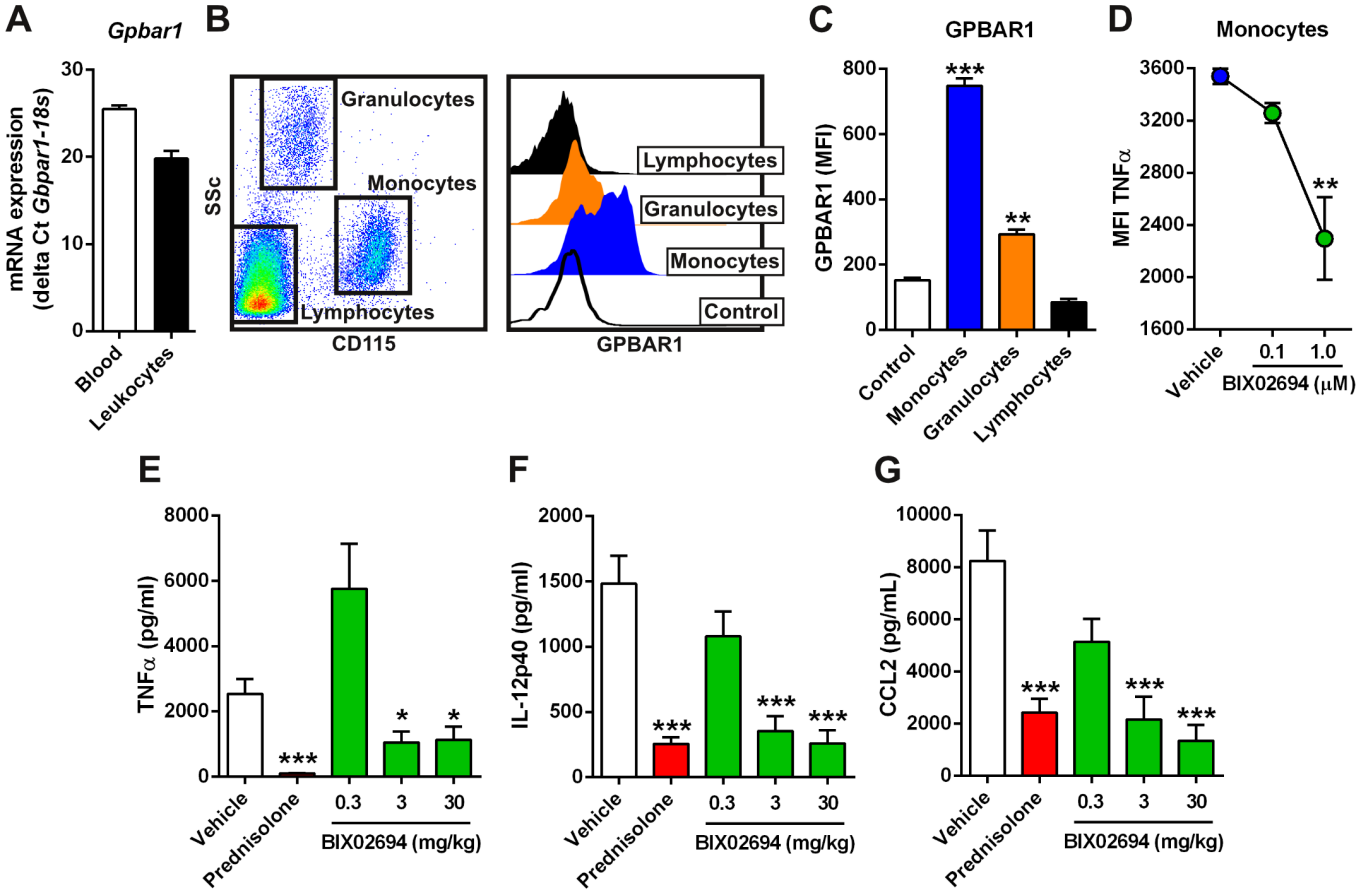
Mouse monocytes express GPBAR1 and its agonism reduces LPS-induced cytokine production. (A) *Gpbar1* and *18s* mRNA levels were assessed by Taqman in C57BL/6 mouse whole blood and isolated leukocytes (n = 2 for leukocytes, n = 5 for blood). (B–C) GPBAR1 protein levels were assessed on mouse blood cells by flow cytometry using CD115 to stain monocytes. (B) Gating scheme and representative histograms of lymphocytes, granulocytes and monocytes are shown (n = 1 is shown, representative of 3 mice). (C) Median fluorescence intensity of GPBAR1 on monocytes, granulocytes, and lymphocytes is shown (n = 3, averaged). Monocytes stained with an isotype control are the control sample (B) and (C). (D–F) Impact of GPBAR1 agonism on LPS induced cytokines. Blood from C57BL/6 mice (n = 5, averaged) was stimulated ex vivo with 100 ng LPS in the presence of BIX02694 and intracellular TNFα in monocytes was measured by flow cytometry (D). Balb/c mice were challenged with 2 µg LPS, in the presence of vehicle, Prednisolone (3 mg/kg), or BIX02694 (0.3, 3, 10 mg/kg) and serum cytokine levels were measured; TNFα at 1 hour (E) IL-12p40 (F) and CCL2 (G) both at 4 hours post LPS administration.

GPBAR1 agonism with our selective tool molecules reduced proinflammatory cytokine production from activated human monocytes and macrophages. To determine if similar mechanisms would occur in mouse monocytes, we first assessed *Gpbar1* mRNA levels in both whole blood and isolated leukocytes from C57BL/6 mice. *Gpbar1* was expressed and its expression was increased in the isolated leukocytes (Fig. 4A). We next assessed GPBAR1 protein expression on circulating blood cells and found that its expression was highest on monocytes. Lower levels were also detected on granulocytes, but not on lymphocytes (Fig. 4B, C). To determine if agonism of GPBAR1 would also restrict mouse monocyte cytokine production in response to LPS, we isolated mouse blood and stimulated the cells with 100 ng LPS ex vivo in the presence of BIX02694 (at 0.1 and 1 µM) and performed intracellular staining for TNFα after 5 hours. Agonism of GPBAR1 resulted in a significant reduction in TNFα production from monocytes (Fig. 4D). These data demonstrate that BIX02694 cross reacts with mouse GPBAR1 and can impact monocyte response to LPS.

To understand if BIX02694 is pharmacologically active in vivo and if agonism of GPBAR1 would also reduce LPS-induced proinflammatory cytokine production in vivo, Balb/c mice (due to their more consistent response to LPS) were dosed with BIX02694 at 0.3, 3, and 30 mg/kg (10 mice per group) or prednisolone (3 mg/kg) and subsequently challenged with LPS at 2 µg per mouse. Serum cytokine levels of TNFα (1 hour post-challenge), IL-12p40 (4 hours post-challenge), and CCL2 (4 hours post-challenge) were assessed and found to be significantly decreased in a dose-dependent manner with GPBAR1 agonism (Fig. 4E–G). Prednisolone is included as a positive control in the experiments as it is a steroid which has strong anti-inflammatory properties and blocks all LPS-mediated responses in all immune cells. These data demonstrate that BIX02694 is pharmacologically active in vivo.

**Figure 5:**
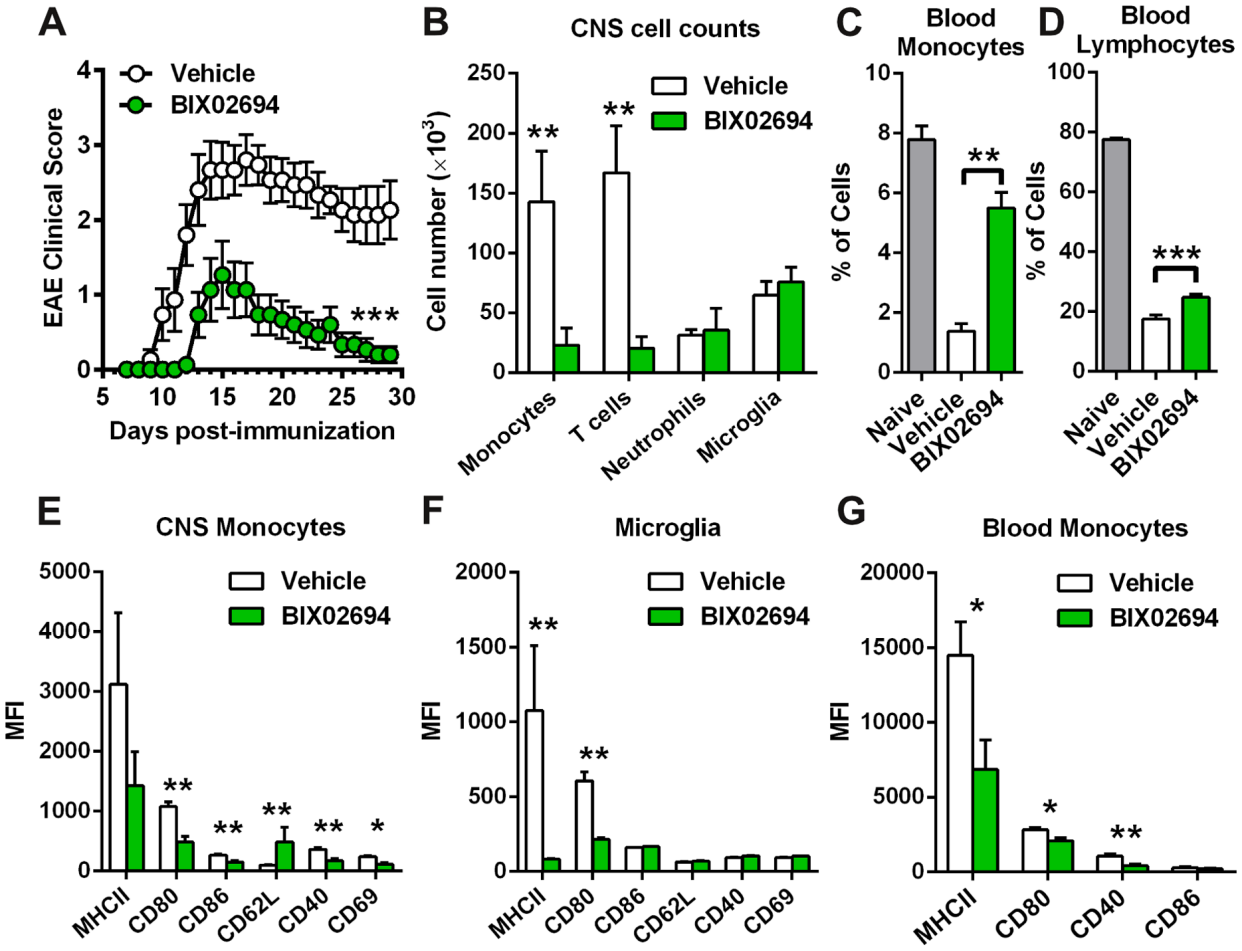
GPBAR1 agonism reduces EAE disease severity. (A) EAE clinical scores are shown for both vehicle and BIX02694 groups (n = 10 mice per group). (B) Cell counts of CNS immune cells assessed on day 13 post-immunization are shown (n = 5 mice). (C) Percentages of blood monocytes (n = 5) or (C) lymphocytes (n = 5) are shown for naive mice (n = 8), along with EAE mice treated with either vehicle or BIX02694. Protein expression of activation markers on both CNS-infiltrating monocytes (E) and microglia (F) are shown (n = 5 mice per group). (G) Protein expression of activation markers on blood monocytes from EAE mice treated with either vehicle or BIX02694 (n = 5 mice per group).

Given the ability of BIX02694 to act as a GPBAR1 agonist to reduce monocyte activation in vitro and proinflammatory cytokine production in vivo, we tested whether this GPBAR1-mediated effect on monocyte activation is sufficient to impact an autoimmune disease in a more chronic setting, as it may have therapeutic implications for human diseases. We chose the mouse model of EAE using C57BL/6 mice where inflammation is triggered in the CNS by rupturing the blood brain barrier using pertussis toxin together with immunization with myelin peptide in the presence of adjuvant. Both the initiation and continuation of inflammation is dependent on both activation of microglia in the CNS and monocyte recruitment and activation in the periphery and in the CNS. This in turn, further activates adaptive immune responses (particularly T cell recruitment and reactivation in the CNS). Together, these activities lead to the observed symptoms of progressive paralysis (observable starting on approximately day 7, reaching a peak at day 14 after which the disease slowly starts to resolve over the next 2–3 weeks). Mice were dosed with the GPBAR1 agonist, BIX02694, twice a day at 30 mg/kg starting before immunization and continued for 30 days (EX00000246 does not have sufficient systemic exposure for application in these studies). Animals were evaluated daily for signs of disease progression and clinical scores. Mice treated with BIX02694 had significant reductions in the clinical score both at peak of disease and at the termination of the study (Fig. 5A).

A significant aspect of the EAE model is a profound influx of cells from the periphery into the CNS during the course of the disease. Since we have demonstrated modulation of CCL2, along with other chemokines in monocytes, we hypothesize that agonism of GPBAR1 may block immune cell trafficking to the CNS. We therefore evaluated monocyte and T cell numbers in the CNS at the peak of disease where their numbers are at their highest (at disease scores of 2 and 3, data not shown and [22]) (Fig. 5B) and found a highly significant reduction (84% & 88%, respectively). This reflects a specific impact on these cells, since the number of neutrophils and microglia in the CNS was not affected (Fig. 5B). Given the reduction in monocyte infiltration into the CNS with GPBAR1 agonism, we assessed if this may be due to a reduction in blood monocytes. We analyzed the level of blood monocytes during EAE and, as expected, we observed a significant reduction in blood monocytes that corresponds with significant increases in CNS monocytes (Fig. 5C). However, mice treated with BIX02694 had significantly more circulating monocytes than the vehicle treated group, with levels almost reaching those of naive mice suggesting that the reduction in CNS monocytes is not due to lack of blood monocytes, but rather due to lack of trafficking to the CNS. Blood lymphocytes are also significantly reduced with disease and GPBAR1 agonism lead to a slight (but significant) normalization in numbers (Fig. 5D). This likely reflects indirect effects on T cells as they do not express GPBAR1.

Given the impact of GPBAR1 agonists on monocyte activation in vitro, we determined their activation status of monocytes and microglia in vivo in the CNS and the activation status of monocytes in the peripheral blood. We assessed the level of surface proteins that are known to be upregulated (MHCII, CD80, CD86, CD40, and CD69) or downregulated (CD62L) upon myeloid cell activation. With GPBAR1 agonism, we observed significant decreases in most of these activation markers on CNS monocytes including CD80, CD86, CD40, and CD69. Consistently, CD62L was increased on monocytes with GPBAR1 agonism (Fig. 5E). On microglia from the CNS, the two activation markers that are significantly upregulated with disease are MHCII and CD80 and both of these are reduced with GPBAR1 agonism (Fig. 5F). We also measured these activation markers on blood monocytes and found significant reductions in MHCII, CD80, and CD40, similar to monocytes in the CNS (Fig. 5G). These data demonstrate that agonism of GPBAR1 reduces the numbers of monocytes and T cells in the CNS, and reduces monocyte and microglia activation in the CNS and monocyte activation in the periphery. Together this results in reduced EAE disease severity as measured by clinical score.

## Discussion

The significance of GPBAR1 signaling in metabolism and glucose homeostasis has been demonstrated in numerous publications, as reviewed recently [1]. However, its potential role in inflammation is not well understood, although some effects on macrophages have recently been described [5]–[9]. In this report, we identify and use highly selective GPBAR1 agonists and complement them with the natural GPBAR1 agonist, Lithocholic acid in vitro, to demonstrate that GPBAR1 agonism reduces proinflammatory responses in human monocytes in vitro and that GPBAR1 agonism is sufficient to reduce autoimmune/inflammatory responses in vivo in mouse. Our hypothesis is that reduction in inflammation in vivo is mediated by direct effects of GPBAR1 on blocking myeloid cell activation. To support this concept of reduction in inflammation, we show direct effects on monocyte activation in peripheral blood which can, in turn, impact monocyte trafficking to sites of inflammation. Also, we show reduced monocyte and microglial activation at the site of inflammation. This reduced local activation of myeloid cells would be expected to result in reduced production of inflammatory mediators and failure to reactivate T cells, thus blocking local T cell proliferation in the CNS. Together these observations suggest that TGR5 agonism is a novel way to reduce the cycle of inflammation and may offer a novel therapeutic approach for inflammatory indications.

We confirm the potency of our molecule by demonstrating effects of GPBAR1 agonism on cAMP induction and on cytokine reduction in response to LPS, as previously described [5]–[9]. In addition, we demonstrate that GPBAR1 agonism has an impact in blunting responses to other TLR agonists in addition to TLR4. While we see that GPBAR1 agonism can block responses to an array of TLR agonists, the blockade is quite selective as only a subset of LPS induced proteins and genes are altered in monocytes when treated with GPBAR1 agonist. At the gene level we observed that agonism of GPBAR1 leads to decreased IL-12 expression and increased IL-10 expression after LPS stimulation, similar to published reports with bile acids (GPBAR1 agonists) in mouse and human [6], [23]. GPBAR1 also regulates production of other inflammatory mediators (e.g. VEGFA, OSM), chemokines (e.g. CXCL6, CCL1, 2, 8, 18 and 22), CCR7 chemokine receptor and surface markers of monocyte activation (e.g. TNFSF15 and CD70).

Importantly, we found that selective agonism of GPBAR1 alters cytokine production in response to LPS activation in vivo and alters monocyte activation in the periphery and in the CNS. GPBAR1 agonism also alters microglia activation during inflammation in the brain. Our results in the EAE model are consistent with a report that oleanolic acid (a GPBAR1/FXR agonist) can reduce disease severity in EAE [14]. Although the authors did not attribute the effects to GPBAR1 activity on monocytes in their paper, they did note that TNFα and osteopontin in the tissue and TNFα, MIP-1α, IFNγ, and MCP-1 in serum was reduced, which is consistent with an impact on myeloid cell activation. Similarly, McMahan et al. [23] demonstrated that a dual GPBAR1/FXR agonist increased the proportion of Ly6C-low monocytes and increased the proportion of genes associated with an alternatively activated macrophage phenotype such as IL-10. Our data suggests that these effects are mediated through GPBAR1 agonism.

The EAE model is a useful system to explore the impact of myeloid cell activation in vivo as myeloid cells have a key role in development of inflammation and the associated tissue damage: microglia activation is the first and critical step [24], [25], followed by monocyte activation [25]–[27] and monocyte trafficking to the CNS (CCL2) [22], [28], [29]. This in turn affects T cell recruitment; specific examples include T cell infiltration into the CNS is blocked during EAE in CCR2^−/−^ mice that have reduced monocyte numbers and reduced monocyte trafficking to the CNS [30] and CXCL10 production by monocytes is thought to lead to T cell recruitment [25].

We have shown that agonism of GPBAR1 has a key impact on myeloid cell activation; reduced production of inflammatory mediators and chemokines (such as CCL2 production) by myeloid cells, reduction in microglia activation, reduction in monocyte activation and expression of molecules required for trafficking by monocytes (CCR7). Together these data offer a molecular explanation for our finding that GPBAR1 agonism in EAE reduced inflammation and reduced the number of CNS-infiltrating monocytes and T cells. Although we have monitored this phenomenon only at the peak of the disease, the data are consistent with this molecular signature. We conclude that the normal monocyte numbers in the periphery reflect both the reduced chemotaxis signal from microglia in the CNS and reduced direct monocyte activation in the periphery. In addition to a critical role for myeloid cells in EAE, local T cell reactivation is also an important process in driving pathogenesis [31]. We conclude that the reduced numbers of T cells in the periphery with GPBAR1 agonism suggests that T cells can still traffic to the CNS, but they fail to be locally reactivated by antigen presenting cells such as microglia and monocytes due to the reduced activation state and antigen presenting capacity of the myeloid cells. Therefore, T cells don't proliferate and their numbers in the brain remain low. The fact that GPBAR1 agonism does not result in a complete reduction in inflammation may be due to neutrophil infiltration into the CNS since we do not observe an impact on neutrophil infiltration. These neutrophils might be the source of the T cell trafficking signal.

Our hypothesis, therefore, is that GPBAR1 agonism reduces microglia and monocyte activation and this reduced myeloid cell activation leads to the other observed downstream effects in EAE. It is these downstream effects on monocytes and T cell activation that translate into reduced disease severity. This hypothesis is consistent with published studies demonstrating that GPBAR1 agonism reduces adhesion of monocytes to endothelial cells [8], and with our in vitro studies on myeloid cells and by expression studies demonstrating that GPBAR1 is found at higher levels on monocytes than granulocytes and lymphocytes. Furthermore, we did not observe any impact of GPBAR1 agonists on other primary human cells (in assays measuring activation of B cells or T cells, data not shown).

These data demonstrate the effects of selective GPBAR1 agonism on monocytes and strengthen the rationale for GPBAR1 agonists as potential therapeutic agents in autoimmune diseases. Given its restricted expression profile, it is attractive to suggest that it could be a beneficial therapy for the treatment of inflammatory disorders, particularly if they are associated with metabolic syndrome/obesity. However, recent reports suggest that GPBAR1 agonism also has profound effects on blood pressure, which may preclude the use of current systemically available agonists as therapies for inflammatory disorders [32]–[34]. Unconventional pharmacological approaches will need to be taken to deliver the compounds to the key site of action to maximize the potential benefits and reduce the potential effects on blood pressure in order to develop a successful therapeutic.
